# Photothermally driven fast responding photo-actuators fabricated with comb-type hydrogels and magnetite nanoparticles

**DOI:** 10.1038/srep15124

**Published:** 2015-10-13

**Authors:** Eunsu Lee, Dowan Kim, Haneul Kim, Jinhwan Yoon

**Affiliations:** 1 Department of Chemistry, Dong-A University, 37 Nakdong-Daero 550 Beon-gil, Saha-gu, Busan, Republic of Korea, 49315

## Abstract

To overcome the slow kinetics of the volume phase transition of stimuli-responsive hydrogels as platforms for soft actuators, thermally responsive comb-type hydrogels were prepared using synthesized poly(*N*-isopropylacrylamide) macromonomers bearing graft chains. Fast responding light-responsive hydrogels were fabricated by combining a comb-type hydrogel matrix with photothermal magnetite nanoparticles (MNP). The MNPs dispersed in the matrix provide heat to stimulate the volume change of the hydrogel matrix by converting absorbed visible light to thermal energy. In this process, the comb-type hydrogel matrix exhibited a rapid response due to the free, mobile grafted chains. The comb-type hydrogel exhibited significantly enhanced light-induced volume shrinkage and rapid recovery. The comb-type hydrogels containing MNP were successfully used to fabricate a bilayer-type photo-actuator with fast bending motion.

Stimuli-responsive hydrogels can undergo reversible volume phase transitions in response to environmental changes, making them highly interesting research targets^1^,^2^,^3^,^4^,^5^,^6^,^7^,^8^,^9^,^10^,^11^,^12^,^13^,^14^,^15^,^16^,^17^. The responsive behavior of hydrogels has been exploited in chemical and mechanical systems, including on-demand drug delivery platforms^3^,^4^, microfluidic valves^5^,^6^, and soft actuators^7^,^8^,^9^,^10^,^11^,^12^,^13^,^14^. The fabrication of hydrogel actuators that undergo reversible and repeatable mechanical movement triggered by external stimuli such as temperature^7^,^8^, pH^9^,^10^, magnetic/electric filed^11^, and light^12^,^13^,^14^ is of particular interest. Because abrupt volume changes of hydrogels can induce movement of deformed actuators, control of the mechanical actions of soft actuators can be achieved with stimuli-responsive hydrogels. Reversible bending of a hydrogel bigel strip comprising temperature-responsive poly(*N*-isopropylacrylamide) (PNIPAm) and non-responsive polyacrylamide (PAAm) has been demonstrated^7^. Temperature-dependent shrinkage of the PNIPAm layer induced bending of the strip toward the shrunken layer. pH-responsive actuators based on polyelectrolytes^9^,^10^, electrically sensitive actuators^11^, and light-triggered actuators^12^,^13^,^14^ have also been reported; nevertheless, the limited rates of swelling and deswelling limited the practical applications of these hydrogel actuators^18^. Swelling and deswelling of hydrogels are dominated by relatively slow collective diffusivity of the polymer network, and the response rate is inversely proportional to the square of the smallest dimension of the gel^19^. Maximizing the rate of the volume change is vital for widespread application of hydrogel actuators.

Motivated by the enhanced swelling and deswelling rates of the hydrogels achieved with comb-type architecture reported in previous studies^20^,^21^,^22^,^23^, we pursued the preparation of hydrogel actuators containing mobile graft chains to overcome the slow actuation kinetics. Previously, we found that magnetite (Fe_3_O_4_) nanoparticles (MNPs) loaded into the temperature-responsive PNIPAm matrix (PNIPAm/MNP) can absorb visible light and convert photo energy into thermal energy, thereby heating the matrix and inducing volume shrinkage of the hydrogel^6^,^15^,^16^. By loading MNPs within the grafted PNIPAm hydrogel (*g*-PNIPAm) matrix, fast responding light-responsive hydrogels were generated.

Here, we note that many studies have demonstrated that the swelling and deswelling rates of the hydrogels could be accelerated by introducing a porous structure through gas-evolution reaction^24^ or freezing polymerization^25^. In addition, the phase-separation method during polymerization under mixed solvent system, which resulted in generation of heterogeneous structure, has been also reported to enhance the swelling kinetics^26^. However the former strategies were limited by poor mechanical property due to the porous structure, while the latter strategy is not feasible to prepare composite due to the use of organic solvent that may precipitate the MNP dispersed in water.

In light of the enhanced response of the grafted hydrogel system containing MNP, we fabricated photothermally driven bilayer-type photo-actuators that show reliable bending motion with maximized volume change and minimized response time.

## Results and Discussion

**Figure 1:**
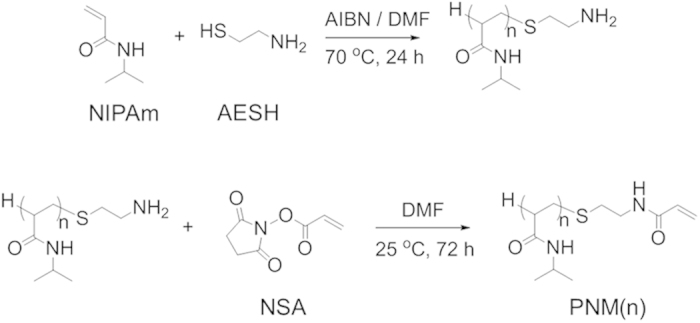
Synthesis of PNIPAm macromonomer through free-radical polymerization.

Fast responsiveness due to changes in the molecular structure of the hydrogel matrix is achieved by introducing graft chains via polymerization of PNIPAm macromonomers (PNM)^20^,^21^. To synthesize PNM, NIPAm was first polymerized in *N*,*N*-dimethylformamide (DMF) at 75 °C for 24 h using 2,2-azobisisobutyronitrile (AIBN) as an initiator and 2-aminoethanethiol (AESH) as a chain transfer agent. (Figure 1) AIBN is well-known thermal initiator for radical polymerization, which is commonly used under moderate heating at 50 ~ 75 °C for effective generation of radical species. The degree of polymerization (DP) was controlled by varying the molar ratio of NIPAm to AESH, yielding amino-terminated PNMs of various molecular weights. Next, amino-terminated PNM was reacted with *N*-acryloxysuccinimide in DMF at 25 °C for 72 h to convert the amino end group to the vinyl group. Thus, PNM with a vinyl end group that can participate in the radical crosslinking process was successfully synthesized. (Figure 1) The DP of PNM was determined via NMR analysis; the number-average molecular weights were measured by gel permeation chromatography.

Light-responsive comb-type *g*-PNIPAm was prepared by free radical polymerization of the pre-gel solution containing macromonomer PNM, *N*,*N*-methylene bisacrylamide as a crosslinker, and MNP, thereby entrapping MNP within the PNIPAm matrix after polymerization. Consequently, the linear PNIPAM chains could be grafted onto the crosslinked PNIPAm network by fixing one end, providing free mobile chains that promote conformational change. *g*-PNIPAm samples prepared with PNM of varying DP (*n*) are designated as *g*-PNIPAm(*n*). The superparamagnetic MNPs used in the work were prepared by co-precipitation method and followed by two-step addition of primary and secondary surfactants to prevent the aggregation of the nanoparticles and disperse in water^6^. The average diameter of MNPs is found to be 12.5 nm by analyzing TEM image. (Figure S1).

**Figure 2:**
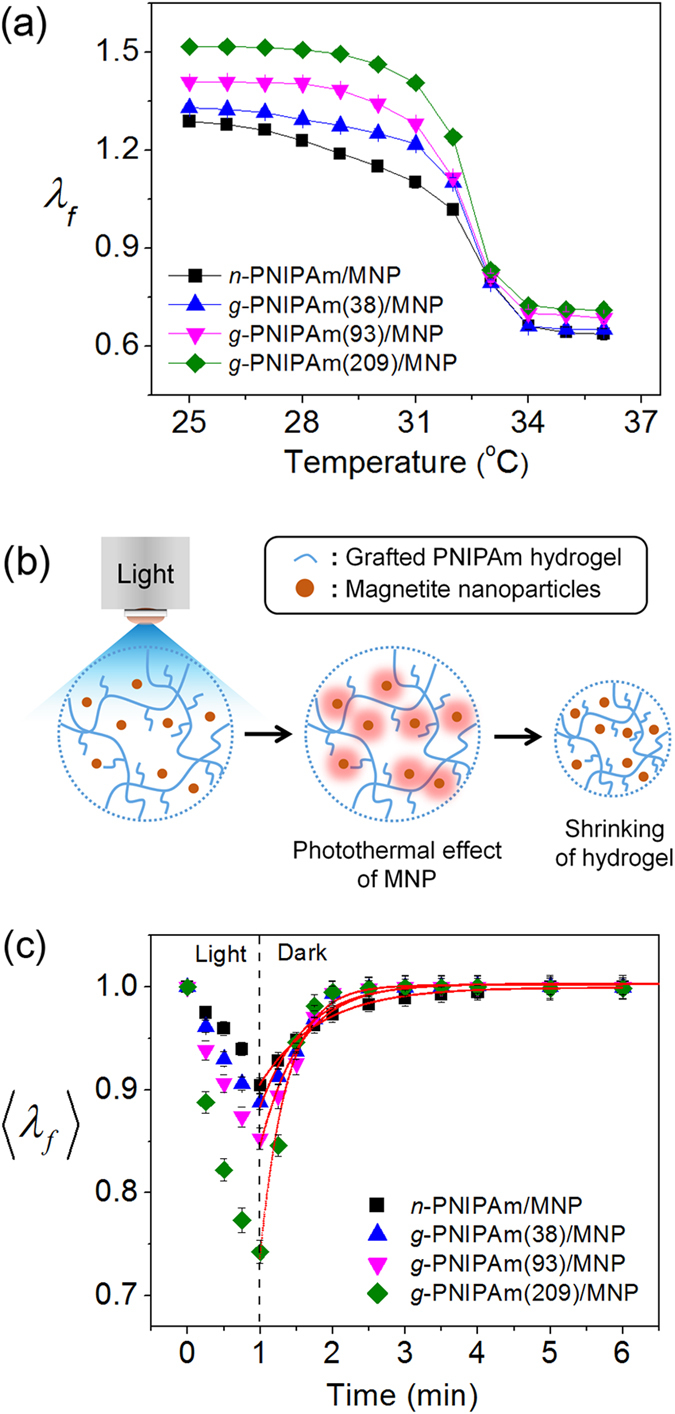
(**a**) Temperature-dependent linear swelling ratio (*λ*_*f*_) during heating run for normal PNIPAm (*n*-PNIPAm) and grafted PNIPAm hydrogels (*g*-PNIPAm) with various degrees of polymerization (DP) of PNIPAm macromonomers. (**b**) Schematic illustration for the volume shrinking of the grafted hydrogels containing MNPs under irradiation of light. (**c**) Normalized linear swelling ratio <*λ*_*f*_> for *n*-PNIPAm and *g*-PNIPAm containing magnetite nanoparticles (MNPs) exposed to visible light (41.8 mW/cm^2^) for 1 min at 25 °C. The red line is the fitting result from equation (1).

The temperature dependent linear swelling ratios (*λ*_*f*_), defined as the extent to which the gel swells in each dimension in the equilibrium state when immersed in water, were determined for *g*-PNIPAm(*n*). Parameter *λ*_*f*_ was quantified by increasing the temperature in increments of 1–2 °C over 10 min, followed by holding the temperature constant for an additional 10 min prior to determining the degree of swelling. The thermal response of *g*-PNIPAm/MNP and the normal crosslinked PNIPAm hydrogel (*n*-PNIPAm) were comparable. (Figure 2a) The *n*-PNIPAm and *g*-PNIPAm hydrogels underwent phase transition at 32 °C, indicating that grafting of PNIPAm has little influence on the thermo-responsivity of the composite hydrogels. The *g*-PNIPAm hydrogels exhibited a higher swelling ratio than *n*-PNIPAm. The *λ*_*f*_ values of the *g*-PNIPAm(*n*) samples ranged from 1.32 to 1.51 depending on the DP of PNM (Table 1), while *λ*_*f*_ of *n*-PNIPAm with an otherwise identical composition was 1.29. The increased swelling ratio of the grafted hydrogels may result from increased hydration due to chain expansion of the free grafted chains.

A volume phase transition is expected upon illuminating the thermally responsive hydrogel network containing photothermal materials with visible light. We found that the temperature of the 2.3 wt% of aqueous MNP dispersion increased ~7 °C over 1 min under irradiation with visible light of 41.8 mW/cm^2^, indicating that sufficient heat is produced under visible light irradiation to cause a substantial temperature increase through photothermal conversion of MNP. (Figure S2) MNPs entrapped in the thermally responsive PNIPAm hydrogel matrix absorb visible light and generate heat, thus triggering a volume shrinking of the thermally responsive hydrogel matrix (Fig. 2b).

The normalized change in the linear swelling ratio (<*λ*_*f*_>) for *g*-PNIPAm/MNP samples of various DP under visible light irradiation for 1 min was evaluated. The measured linear swelling ratios were normalized by the swelling ratios of the gels in the equilibrium swelling state before illumination. The hydrogels containing MNP underwent significant volume changes, depending on the DP of PNM (Fig. 2c). Exposure to blue light (41.8 mW/cm^2^) for 1 min induced a 0.7% change of <*λ*_*f*_> for *n*-PNIPAm, while the corresponding values for the *g*-PNIPAm samples ranged from 11% to 23% in proportion to the DP of PNM. Irradiation of grafted *g*-PNIPAm(209) led to a decrease in the <*λ*_*f*_> to 0.77, corresponding to a 57% decrease in the volume compared to the initial equilibrium state before irradiation. The increased light-induced volume shrinkage resulted from rapid shrinkage of the hydrogel due to the enhanced mobility of the graft chains. During shrinkage, hydrophobic aggregates were readily formed due to the mobile graft chains that are not entrapped in the network structure of the backbone.

We revealed that 2.3 wt% is optimal concentration of MNP for maximum volume change, since increase of the MNP concentration offset the light transmittance, resulting the saturation of photothermal efficiency. (Figure S4 and S5).

Shortly after discontinuing visible irradiation, the hydrogels recovered the original volume by absorbing water. Compared to the gradual increase of the swelling ratio of *n*-PNIPAm/MNP to its former state within 4–5 min after discontinuing irradiation, *g*-PNIPAm/MNP exhibited significantly enhanced swelling kinetics in proportion to the DP of PNM. For quantitative analysis of the reswelling rate of the composite hydrogels, the reswelling profiles were fitted to the swelling kinetics equation reported by Tanaka^19^. Based on the Li–Tanaka formalism for the swelling kinetics of a disc-shaped hydrogel, the swelling kinetics can be expressed in terms of single exponential decay with time as follows:


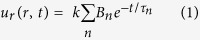


where *k* is proportional factor, *B*_*n*_ is related to the corresponding eigenfunction evaluated at the boundary condition, and τ_n_ is the characteristic time of swelling, which is proportional to the square of the hydrogel thickness and is inversely proportional to the collective diffusion coefficient *D*. By fitting the reswelling profiles using equation (1), the *τ*_1_ values were obtained. (Table 1) The *τ*_1_ value for *n*-PNIPAm was 0.82 compared to 0.62 for *g*-PNIPAm(38). Preparing the hydrogel with *g*-PNIPAm(209) increased *τ*_1_ to 0.38. Thus, τ_1_ increased as the DP of PNM increased, meaning that the reswelling kinetics is enhanced for the grafted hydrogel network. The *g*-NIPAm sample prepared with PNM having a DP of more than 500 had a *τ*_1_ value similar to that of *g*-PNIPAm(209), indicating that no more enhancements on swelling kinetics were achieved by increasing the DP of PNM. The fast rate of volume recovery results from the free and mobile graft chains in the hydrogel network. The graft chains promote conformational changes of the hydrogel owing to the enhanced mobility of the polymer network.

**Figure 3:**
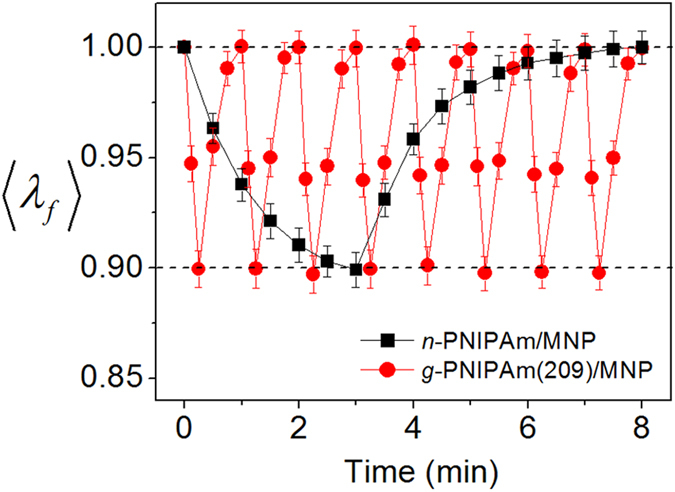
Normalized linear swelling ratios <λ_*f*_> for n-PNIPAm and g-PNIPAm(209) containin*g* MNP exposed to visible light (41.8 mW/cm2) at 25 °C. Dotted lines are drawn at <*λ*_*f*_> = 1.0 and 0.9.

To compare the responsiveness of *g*-PNIPAm and *n*-PNIPAm, the photo-induced changes of <*λ*_*f*_> between 0.9 and 1.0 were observed. Both hydrogels were irradiated with visible light until *λ*_*f*_ decreased by ~10%, corresponding to a volume change of 30%. Irradiation for 3 min led to an 10% decrease of <*λ*_*f*_> for *n*-PNIPAm, while irradiation for 15 s was sufficient to induce a comparable decrease for *g*-PNIPAm(209). (Figure 3) Moreover, *g*-PNIPAm(209) recovered its original volume within 1 min after discontinuing irradiation, while the *n*-PNIPAm hydrogel gradually reverted to the initial state over 4–5 min. Thus, with the grafted structure, the photo-irradiation and recovery times could both be minimized to achieve a targeted volume decrease relative to *n*-PNIPAm under identical experimental conditions. Conclusively, 8 cycles of photo-induced volume change could be achieved for *g*-PNIPAm(209) during the time required for 1 deswelling-swelling cycle of *n*-PNIPAm.

**Figure 4:**
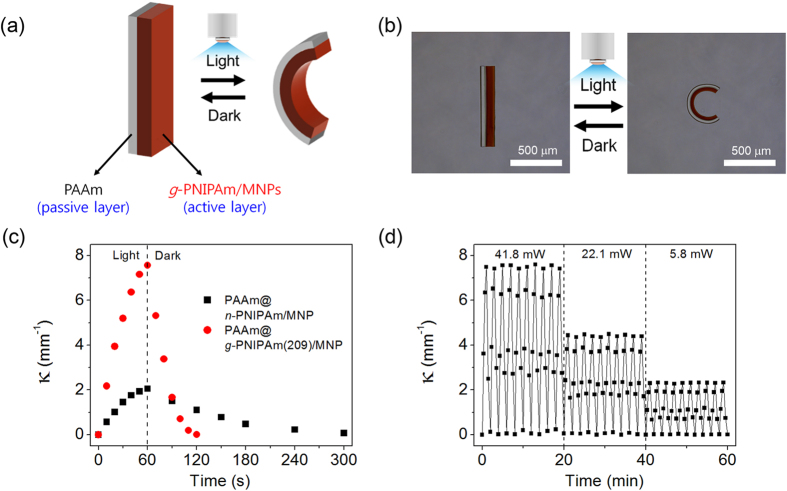
Bending motion of the bilayer-type actuator comprising poly(acrylamide) (PAAm) and *g*-PNIPAm(209)/MNP under visible light irradiation. (**a**) schematic illustration of the bilayer-type actuator; (**b**) micrographs for the bilayer actuator before (left) and after (right) irradiation with 41.8 mW/cm^2^ of visible light for 1 min at 30 °C; (**c**) curvature (κ) changes of the actuators; (**d**) empirically determined κ for the bilayer actuator comprising PAAm and *g*-PNIPAm(209)/MNP recorded for 30 cycles under visible light irradiation with various intensities (1 cycle: irradiation with visible light for 60 s followed by removal from light source for a further 60 s).

Based on the improved light responsiveness and swelling rate of the grafted PNIPAm, we fabricated a light-driven bilayer-type actuator that can rapidly undergo bending motions by combining *g*-PNIPAm(209)/MNP as an active layer and polyacrylamide (PAAm) as a passive layer. The dimensions of the *g*-PNIPAm(209)/MNP layer change reversibly in response to photo-irradiation, while the PAAM layer retains its dimensions. Since the passive layer of PAAm restricts dimensional changes of the active layer of *g*-PNIPAm(209)/MNP in one direction, the size mismatch induces folding of the bilayer in one direction due to the stress at their common boundary, as illustrated in Fig. 4a.

The hydrogel bilayer consisted of a PAAm layer and a *g*-PNIPAm(209)/MNP layer prepared in sequence by radical polymerization. First, a *g*-PNIPAm(209)/MNP sheet was synthesized between two glass coverslips separated by 60 μm spacers. After gelation of the first hydrogel layer, the top coverslip was detached from the hydrogel sheet and extra spacers with a thickness of 30 μm were also placed on the existing spacers and the coverslip was replaced. Next, a pre-gel solution of PAAm was filled into the space above the existing *g*-PNIPAm(209)/MNP sheet and polymerized for 1 h. Finally, the covalently combined bilayer of *g*-PNIPAm(209)/MNP and PAAm was obtained. After cutting the bilayer into a stick shape, the bilayer was swollen in water.

Changes in the shape of the bilayer actuator in response to visible light irradiation for 1 min were monitored. To maximize the bending motion in response to photo irradiation, the temperature of the medium was set to 30 °C, which is just below the lower critical solution temperature of *g*-PNIPAm^6^. As seen in Fig. 4b, the fully swollen bilayer actuator was straight when in the equilibrium state before exposure to light. Exposure to visible light (41.8 mW/cm^2^) for 1 min induced bending of the bilayer actuator toward the *g*-PNIPAm(209)/MNP side. The *g*-PNIPAm(209)/MNP layer rapidly shrank in response to light-irradiation, whereas the PAAm layer was insensitive to the light illumination and temperature change. The consequent mismatch of the dimensions resulting from shrinkage of one layer induced bending of the bilayer into an arc shape. The photo-induced actuation between the straight and the arc shape was fully reversible.

The curvature of the bilayer was further evaluated as a function of time and plotted in Fig. 4c. The curvature (κ) of the bilayer actuator is defined as the inverse of the radius of the actuator at a specific stage. When the actuator is nearly straight, κ will be close to zero; κ increases as the actuator changes to the arc shape. Figure 4c show that irradiation of the bilayer actuator fabricated with *g*-PNIPAm(209) with blue light (41.8 mW/cm^2^) for 1 min increased κ to 7.5 mm^−1^ compared to the κ value of 2.0 mm^−1^ for *n*-PNIPAm. Upon discontinuing irradiation, κ of the *n*-PNIPAm actuator slowly decreased to the original value within 4–5 min, whereas the κ value of the *g*-PNIPAm actuator rapidly returned to the initial state within 1 min.

To confirm the reproducibility of the light driven actuation behavior, the actuation movement was successively repeated for 30 cycles by irradiating the bilayer with visible light for 60 s and then discontinuing irradiation for a further 60 s. The hydrogel actuator exhibited a fully reversible bending motion at each cycle with constant and reliable curvature changes. As seen in Fig. 4d, the κ of the actuator increased and quickly returned to its initial value as the light was switched on and off several times. Similar maximum values of the curvature were observed for each cycle under constant light intensity, confirming good reproducibility of the bending motion. Irradiation of the bilayer with light of lower intensities resulted in less bending of the bilayer actuator, indicating that the degree of bending can be controlled by varying the light intensity.

## Conclusion

In conclusion, comb-type grafted hydrogels were successfully prepared by the polymerization of synthesized macromonomers bearing graft chains. The comb-type hydrogels undergo rapid swelling and deswelling due to the free mobile graft chains, providing effective platforms for fabricating hydrogel actuators. By using the comb-type hydrogel matrix with photothermal MNP, the responsiveness and swelling rate of the light-responsive hydrogels could be enhanced. Moreover, we demonstrated the fast bending motion of a bilayer-type photo-actuator made with the developed light-responsive hydrogels. Degree of actuation could be reliably controlled with intensity of the light.

## Methods

### Materials

*N*-isopropylacrylamide (NIPAm), 2-aminoethanethiol (AESH), and *N*-acryloxysuccinimide (NSA) were obtained from Tokyo Chemical Industry (Nihonbashi-honcho, Tokyo, Japan). 2,2′-Azobisisobutyronitrile (AIBN) and diethyl ether were purchased from Samchun Pure Chemical (Pyeongtaek, Gyeonggi-do, Korea). Aqueous fluorescent polystyrene beads of 3 μm were obtained from PolyScience Inc. (Warrington, PA, USA). All other chemicals were obtained from Sigma-Aldrich (St. Louis, MO, USA) and used as received.

### Synthesis of PNIPAm macromonomers (PNM)

The PNIPAm macromonomer was synthesized by a two-step reaction. First, to obtain the amino-terminated PNIPAm macromonomer, 1.28 g (11.3 mmol) of NIPAm and 193 mg (2.5 mmol) of AESH (as a chain-transfer agent) were dissolved in 20 mL of *N,N*-dimethylformamide (DMF). For free radical polymerization, 24 mg (0.16 mmol) of AIBN was added to the solution and the reaction mixture was heated at 75 °C for 24 h under nitrogen atmosphere. After polymerization, DMF was removed by rotary evaporation and vacuum drying. The concentrated solution was dissolved in 10 mL of acetone and then precipitated in diethyl ether. The precipitate was recovered by filtration and vacuum dried. In the second step, the obtained amino-terminated PNM (0.87 g) was reacted with 3.4 g of *N*-acryloxysuccinimide in 50 mL of DMF at 25 °C for 72 h to convert the amino end group to the polymerizable vinyl group. After the reaction, the reaction mixture was dropped into diethyl ether and the precipitate was collected by filtration. After drying under vacuum, PNM was obtained as a white powder.

The spectral data for PNM(n) were as follows: ^1^H-NMR(400 MHz, D_2_O): δ 0.92 (m, 6*n*H), 1.32 (m, 2*n*H), 1.88 (m, 2*n*+1H), 2.76 (d, 2H), 2.91 (d, 2H), 3.74 (s, *n*H), 5.81–6.17 (m, 3H); (*n* = degree of polymerization).

### Preparation of comb-type hydrogels

The comb-type hydrogels containing magnetite nanoparticles (MNP) were prepared by mixing 85 μL of water containing the amounts of monomer and crosslinker shown in Table 2 with 15 μL of an aqueous dispersion of MNP (15 mg/mL). The MNPs were prepared by the co-precipitation method as described in our previous report^6^. To determine the linear swelling ratio of the hydrogel, 3 μm of fluorescent polystyrene beads (average of 10 ~ 20 beads per ~mm^2^) were added to the solution. The mixed solution was polymerized by adding 0.15 μL of *N*,*N*,*N*′,*N*′‐tetramethylethylenediamine (TEMED) and 0.5 μL of 10 wt% aqueous ammonium persulfate (APS). The resulting solutions were immediately loaded into a capillary channel formed with two coverslips separated by spacers of 150 μm. Gelation was carried out in a sealed chamber under a positive pressure of nitrogen for 1 h. After polymerization, the coverslip and spacers were removed, and the hydrogel was immersed in water.

### Fabrication of bilayer-type actuators

A bilayer-type actuator composed of a grafted poly(*N*-isopropylacryamide)(209)/MNP (*g*-PNIPAm(209)/MNP) layer and a poly(acrylamide) (PAAm) layer was prepared by two-step free radical polymerization. The first layer was prepared by the same procedure used for the comb-type hydrogels, as described in the previous section. The thickness of the spacers used for fabrication of *g*-PNIPAm(209)/MNP was 60 μm; *g*-PNIPAm(209)/MNP was assembled by stacking four aluminum foils (15 μm, SK aluminum, Korea). After gelation of the first hydrogel layer for 1 h, the coverslip was removed from the hydrogel composite; the 30 μm aluminum foils were then placed on the existing spacers to obtain the extra space for the second layer. The PAAm hydrogel layer was fabricated with 100 μL of pre-gel solution containing 9.8 mg of AAm and 0.15 mg of BisAA. Free radical polymerization was initiated by adding 0.15 μL of TEMED and 0.5 μL of 10 wt% APS to the degassed pre-gel solution. After gelation, the coverslips and spacers were removed from the hydrogel bilayer composite, and the hydrogel was then swollen in distilled water. The prepared bilayer was cut into dimensions of about 650 μm × 100 μm.

### Measurement

^1^H NMR spectra were recorded with MR400 DD2 (Agilent, USA) spectrometer using D_2_O as the solvent. The molecular weight and polydispersity index (PDI) of PNM were estimated by gel permeation chromatography (GPC) (Waters, column: Shodex KF-802, KF-803). Tetrahydrofuran and polystyrene were used as the solvent and reference.

To measure the swelling ratios of the composite hydrogels, the position of at least 10 fluorescent beads embedded within the hydrogel was tracked as described previously^27^. The change of the linear swelling ratio is identical to the movement of the fluorescent beads. The reported values are the average of the linear expansion determined for each bead, where the uncertainties correspond to the standard deviation. A high‐pressure mercury short arc lamp (EL‐6000, Leica, Germany) supplied blue light irradiation after passing the light through a blue excitation filter (450–490 nm, I3, Leica, Germany); the intensity of the light after leaving the filter was 41.8 mW/cm^2^, as measured using an ACCU‐CAL™ 50‐LED (DYMAX, USA). The hydrogel actuator was imaged using an inverted microscope (DMI3000B, Leica, Germany).

## Additional Information

**How to cite this article**: Lee, E. *et al.* Photothermally driven fast responding photo-actuators fabricated with comb-type hydrogels and magnetite nanoparticles. *Sci. Rep.* **5**, 15124; doi: 10.1038/srep15124 (2015).

## Supplementary Material

Supplementary Information

## Figures and Tables

**Table 1:** Linear swelling ratio (*λ*_*f*_), extent of decrease of *λ*_*f*_ upon irradiation (*Δλ*_*f*_), and characteristic time of swelling (τ) of hydrogels containing MNP.

Sample	*λ*_*f*_	*Δλ*_*f*_^a^	τ
*n*-PNIPAm/MNP	1.29	7%	0.82
*g*-PNIPAm(38)/MNP	1.32	11%	0.62
*g*-PNIPAm(93)/MNP	1.40	14%	0.55
*g*-PNIPAm(209)/MNP	1.51	23%	0.38

^a^Decrease of *λ*_*f*_ induced by irradiation with blue light (41.8 mW/cm^2^) for 1 min.

**Table 2:** Composition of the polymerization for *n*-PNIPAm and *g*-PNIPAm hydrogels.

sample	NIPAm(mg)	PNM(mg)	M_n_ of PNM^a^(g/mol)	PDI	BisAA(μg)
*n*-PNIPAm	8.38	—	—	—	120
*g*-PNIPAm(38)	—	8.497	4,521	1.07	2.9
*g*-PNIPAm(93)	—	8.498	10,742	1.12	1.2
*g*-PNIPAm(209)	—	8.499	24,090	1.15	0.5

^a^Number average molecular weight determined by GPC.
