# Supplementary material for: Photothermally driven fast responding photo-actuators fabricated with comb-type hydrogels and magnetite nanoparticles
Source: Sci Rep. 2015 Oct 13;5:15124. doi: 10.1038/srep15124 (PMC4602301; doi:10.1038/srep15124)
Supplement: Supplementary Information [file srep15124-s1.pdf]

Supplementary information

Photothermally driven fast responding photo-actuators fabricated with comb-type hydrogels and magnetite nanoparticles

*Eunsu Lee, Dowan Kim, Haneul Kim, Jinhwan Yoon**

Department of Chemistry, Dong-A University, 37 Nakdong-Daero 550 Beon-gil, Saha-gu, Busan, Republic of Korea, 604-714

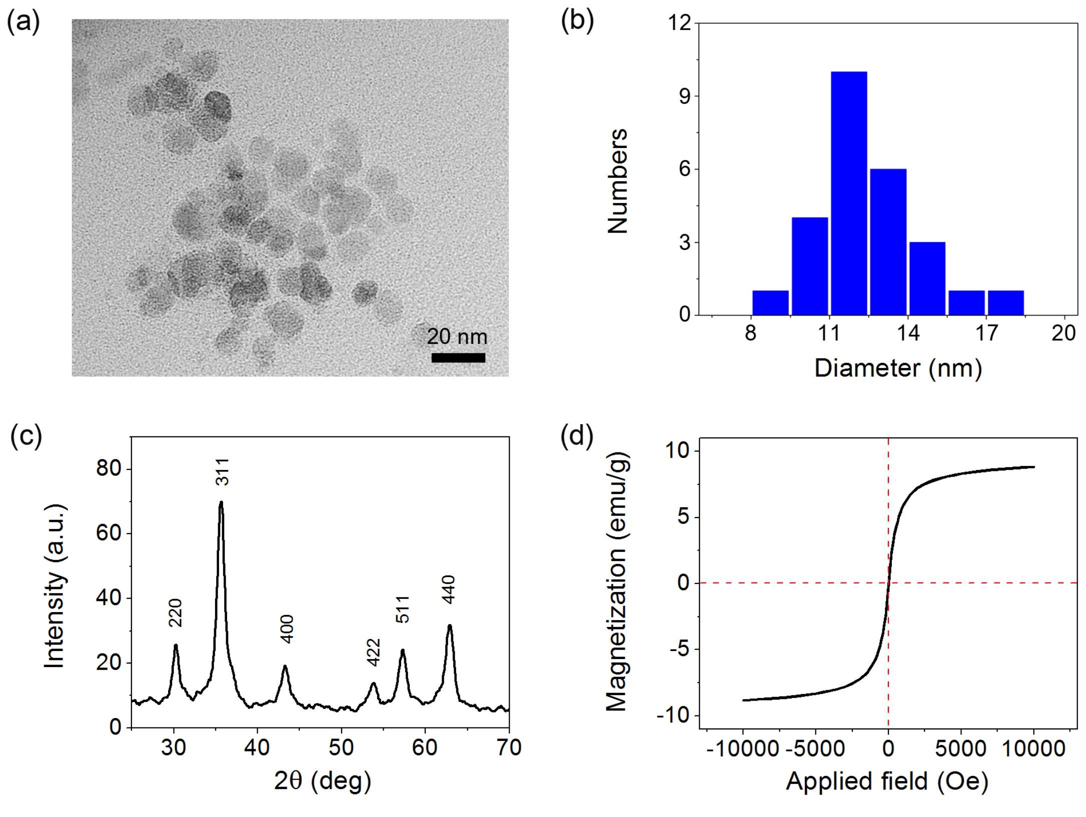

Figure S1. (a) Transmission electron microscopy (TEM) image of MNPs and (b) size distribution determined by analyzing TEM image. (c) X-ray diffraction profile for dried MNPs. (d) Magnetization curve of MNPs measured by superconducting quantum interference device.

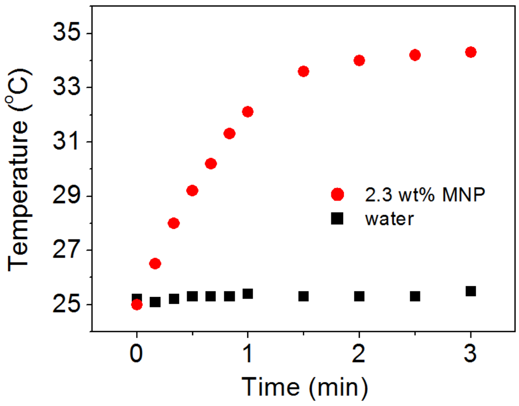

Figure S2. Temperature changes for pure water and 2.3 wt% of aqueous MNP under irradiation with blue light of 41.8 mW/cm^2.

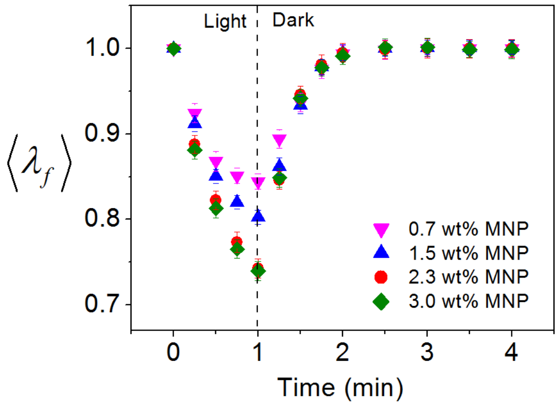

Figure S3. Normalized linear swelling ratio $\langle\lambda_f\rangle$ for *g*-PNIPAm(209) containing 0.7, 1.5, 2.3 and 3.0 wt% of MNP exposed to visible light (41.8 mW/cm^2) for 1 min at 25 °C.

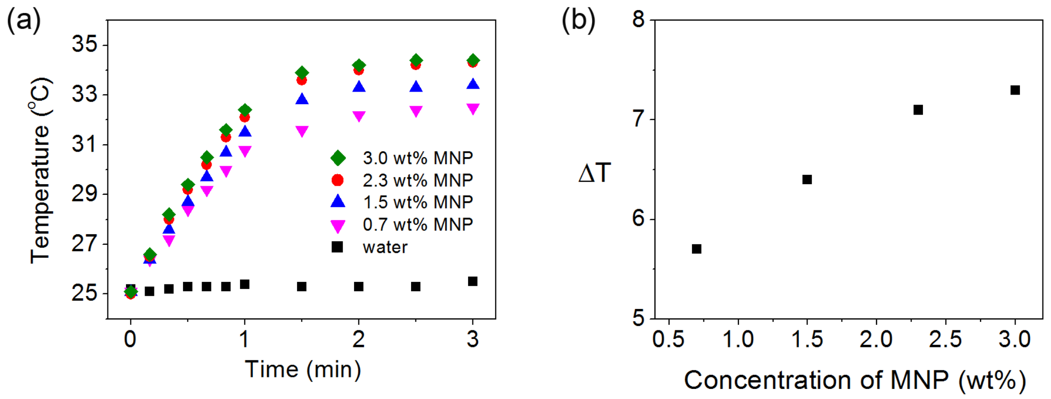

Figure S4. (a) Temperature changes for pure water and 0.7, 1.5, 2.3 and 3.0 wt% of aqueous MNPs under irradiation with blue light of 41.8 mW/cm^2. (b) Degree of temperature increase for 0.7, 1.5, 2.3 and 3.0 wt% of aqueous MNPs under irradiation for 1min.

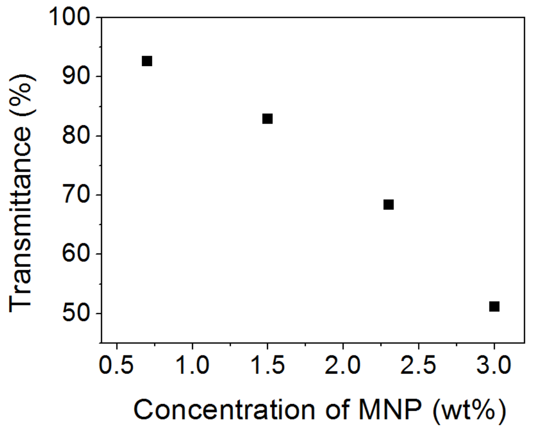

Figure S5. Light transmittances at 450nm for 0.7, 1.5, 2.3 and 3.0 wt% of aqueous MNPs.
